# Configuring green behaviors in hospitality: a drive-state-pressure model analysis of institutional and individual dynamics

**DOI:** 10.3389/fpsyg.2025.1609809

**Published:** 2025-09-05

**Authors:** Jun Zhang, Junbo Cui, Xinchen Chai, Siqi Li

**Affiliations:** School of Humanities and Social Sciences, Beijing Institute of Petrochemical Technology, Beijing, China

**Keywords:** employee green behavior, drive-state-pressure model, fsQCA, institutional pressures, sustainability governance

## Abstract

**Introduction:**

The hotel industry creates significant economic value but also intensifies environmental challenges. Frontline employees’ green behaviors (EGB) are crucial for translating organizational sustainability commitments into practice. This study applies the Drive–State–Pressure (DSP) model to examine how institutional pressures and individual agency jointly shape EGB.

**Methods:**

Survey data were collected from 356 hotel employees in China. Fuzzy-set Qualitative Comparative Analysis (fsQCA) was used to explore configurational pathways leading to task-oriented green behavior (TGB) and voluntary green behavior (VGB).

**Results:**

Coercive, normative, and mimetic pressures promote TGB primarily through compliance, whereas VGB is driven by autonomy and normative alignment. Distinct combinations of pressures and individual states highlight the complex mechanisms underlying EGB.

**Discussion:**

The findings extend institutional theory by bridging macro- and micro-level perspectives through the DSP model and offer practical strategies for sustainability governance. Methodologically, the study demonstrates the value of fsQCA for capturing configurational interactions in employee green behaviors.

## 1 Introduction

The hotel industry, a cornerstone of the global economy (Dutta, 2024), thrives on resource-intensive operations yet faces increasing pressure to adopt sustainable practices. In April 2024, the WTTC projected the sector’s economic impact to hit a record $11.1 trillion. However, in 2019, hospitality and tourism contributed 8–11% of global greenhouse gas emissions—3.9-5.4 billion tons of CO₂ (carbon dioxide, the main greenhouse gas) out of 48.9 billion tons CO₂e (carbon dioxide equivalent, a standard metric including CO₂ and other greenhouse gases; World Travel and Tourism Council, 2024). This dual reality—economic prominence and environmental impact—is under increasing scrutiny. Eco-conscious travelers prioritize sustainability and are willing to pay a premium for certified green accommodations (Velaoras et al., 2025), while investors integrate ESG compliance into investment criteria (Deloitte, 2023). Sustainability efforts ultimately rely on frontline hotel staff, who translate institutional policies into daily practice (Meirun et al., 2024).

Unlike manufacturing, where green behaviors occur backstage, hotel employees enact sustainability in real time—adjusting thermostats, minimizing plastics, and explaining policies—often under guest scrutiny (Li et al., 2020). This creates a sustainability paradox as employees juggle competing priorities: (1) efficiency vs. environment, where energy-saving may delay service (Yenidogan et al., 2021); (2) role ambiguity, with unclear protocols on balancing guest satisfaction and sustainability (Erdogan and Baris, 2007); and (3) motivation fragmentation, where pro-environmental values do not always translate into action (Ruepert et al., 2016). These contradictions expose the limits of frameworks that view green behavior as linear, overlooking the compromises between institutional demands and individual discretion.

Most theories explain employee green behavior (EGB) through individual motivation or structural pressures. The Theory of Planned Behavior (TPB; Khalid et al., 2022) and Social Cognitive Theory (SCT; Zhao and Zhou, 2021) emphasize attitudes and self-efficacy but fail to explain why employees with strong environmental values neglect simple actions (e.g., switching off lights) under work pressure. Even integrative models (Yang et al., 2020) focus too much on psychological factors, overlooking institutional pressures that shape behavior.

Environmental green behavior (EGB) is not a uniform, linear process but an emergent outcome shaped by dynamic interactions between individual agency and organizational pressures. Employee sustainability engagement varies not only in intensity (Ciocirlan, 2023) but also in form (Norton et al., 2015), manifesting as either task-oriented green behavior (TGB)—compliance-driven, role-prescribed actions—or voluntary green behavior (VGB)—discretionary eco-innovation. A key challenge lies in understanding why some employees strictly adhere to corporate sustainability mandates (Sabbir and Taufique, 2022), while others exceed expectations by proactively shaping green initiatives (Ghani et al., 2024).

To address this, the Drive-State-Pressure (DSP) model is introduced. It adapts the Pressure-State-Response (PSR) framework—originally developed in the 1990s and later expanded by the OECD and UNEP—into organizational behavior. The DSP model conceptualizes green behavior as the joint outcome of motivational drivers (Drive), capability conditions (State), and institutional influences (Pressure). In doing so, it provides a dynamic alternative to linear models (Wiernik et al., 2016) and universal assumptions (Norton et al., 2015).

However, prior research on employees’ green behaviors (EGB) has largely relied on linear methods such as regression or SEM. These approaches capture the net effect of single factors but fail to reflect the causal complexity of organizational contexts (Norton et al., 2015; Eva et al., 2017). They overlook equifinality—different configurations of institutional and individual factors leading to similar outcomes—and causal asymmetry, where the presence and absence of outcomes follow different logics (Fiss, 2011). Moreover, linear-additive models implicitly assume that institutional and individual factors exert independent and cumulative effects. Such assumptions overlook the interactive and configurational nature of organizational contexts, where some factors only matter in combination with others or may substitute for one another. To address these limitations, this study employs fuzzy-set Qualitative Comparative Analysis (fsQCA). The fsQCA examines how multiple conditions combine to generate outcomes. By embracing conjunctural causation and asymmetry, it offers a powerful tool for analyzing how institutional pressures and individual agency jointly shape employees’ task-oriented and voluntary green behaviors. The fsQCA examines how multiple conditions combine to generate outcomes, and by capturing conjunctural causation and asymmetry (Xing et al., 2025), it provides a powerful tool for analyzing how institutional pressures and individual agency jointly shape employees’ task-oriented and voluntary green behaviors.

This study contributes to environmental behavior research by developing the Drive-State-Pressure (DSP) model and applying fuzzy-set Qualitative Comparative Analysis (fsQCA). First, it bridges the macro–micro divide in institutional theory by showing how coercive, normative, and mimetic pressures shape task-oriented (TGB) and voluntary green behaviors (VGB). Second, using a configurational approach, it identifies equifinal pathways where institutional forces and individual agency interact via substitution or amplification, challenging linear-additive models. Third, it resolves the compliance-innovation paradox, finding that (1) structured interventions drive TGB but hinder VGB, while (2) flexible institutional frameworks encourage discretionary environmental engagement. These insights advocate a shift from universal policies to context-adaptive strategies that align institutional structures with individual agency to foster sustainable behaviors.

## 2 Theoretical background and literature review

### 2.1 Research on employee green behavior

Employee green behavior (EGB), or employee pro-environmental behavior, refers to workplace actions that benefit the environment (Norton et al., 2015). As a key driver of organizational sustainability, EGB enhances environmental performance and supports sustainable development goals. Scholars often distinguish between two types of EGB: task-oriented green behavior (TGB), which involves environmentally conscious actions embedded in formal job duties such as waste segregation and use of eco-friendly materials (Sibian and Ispas, 2021), and voluntary green behavior (VGB), which encompasses discretionary initiatives such as proposing energy-saving solutions or championing sustainability innovations (Ren et al., 2023). This distinction is critical because TGB is typically compliance-driven, operating within structured control systems and institutional mandates (Norton et al., 2015), whereas VGB is rooted in autonomy and intrinsic motivation. From a managerial perspective, TGB ensures organizational adherence to environmental standards (Zhu et al., 2021), while VGB enables adaptive innovation and long-term sustainability (Zacher et al., 2023). Yet organizations often emphasize compliance through TGB while underutilizing the innovation potential embedded in VGB (Dewiana et al., 2024). Understanding the antecedents of both forms of behavior is therefore essential for balancing compliance enforcement with voluntary engagement.

### 2.2 From PSR to DSP: extending the framework

To theorize the multi-level antecedents of EGB, this study draws on the Pressure–State–Response (PSR) framework, which was first proposed by Canadian researchers in the early 1990s and subsequently developed and widely applied by the Organization for Economic Co-operation and Development (OECD) and the United Nations Environment Program (UNEP) in global environmental assessments. Over the past decades, PSR has been employed in diverse domains such as ecological security evaluation (Zhao et al., 2014), emergency impact assessment (Wei et al., 2012), and sustainable development research (Salemi et al., 2019). Importantly, the PSR framework has not remained static; it has inspired several derivative models, including the Drive–State–Pressure (DSP), Drive–State–Response (DSR), and the Drive–Pressure–State–Impact–Response (DPSIR) frameworks, all of which share a common causal structure for analyzing complex interactions among multiple influencing factors (Jun et al., 2012).

Although PSR originated in environmental science to explain how ecosystems adapt to external pressures (Li, 2004), subsequent studies demonstrate that its logic is not confined to ecological systems. It has also been applied at the individual level—for instance, in explaining psychological or behavioral responses under stress (Hughey et al., 2004)—suggesting its broader generalizability. The underlying logic of “external pressure–internal state–behavioral response” thus provides a transferable framework for analyzing adaptive behavior across domains.

Building on this theoretical commonality, the present study adapts PSR to organizational behavior by proposing the Drive–State–Pressure (DSP) model. This adaptation extends PSR in two key ways. First, the “Response” component is reconceptualized as “Drive,” underscoring that employees are not merely passive reactors but proactive agents whose intrinsic motivation propels environmentally responsible behavior. Second, the DSP model explicitly links institutional pressures (Pressure) with individual capability states (State) and motivational drives (Drive), thereby bridging institutional theory’s macro perspective and organizational behavior’s micro perspective. Within this framework, Drive refers to motivational drivers such as green values (GV), which provide employees with a stable and identity-congruent orientation toward environmental responsibility. State encompasses enabling conditions, including green self-efficacy (GSE)—the confidence to perform environmentally impactful actions—and job autonomy (JA)—the discretion to implement sustainability practices in one’s work. Pressure refers to institutional mechanisms that regulate behavior: green performance management (GPM) as coercive pressure, environment-oriented CSR (ECSR) as normative pressure, and green transformational leadership (GTL) as mimetic pressure. Together, these three dimensions explain how compliance-oriented behaviors emerge under coercive systems, while discretionary innovation is more likely to be fostered when intrinsic motivation and enabling states are strong.

### 2.3 The configurational approach and fsQCA

Traditional variable-centered approaches, such as regression or structural equation modeling, assume that each factor contributes independently and additively to outcomes (Howard and Hoffman, 2018). However, organizational behavior is often the result of conjunctural causation, where outcomes arise from specific combinations of conditions rather than isolated variables. The configurational approach addresses this complexity by emphasizing three key principles: equifinality, whereby different pathways of conditions may lead to the same outcome; causal asymmetry, whereby the conditions associated with the presence of an outcome may differ from those associated with its absence; and holistic combination, whereby conditions derive meaning from their configuration rather than from their independent effects (Fiss, 2011).

Within this paradigm, fuzzy-set Qualitative Comparative Analysis (fsQCA) has emerged as a powerful empirical method. Developed by Ragin, fsQCA operationalizes configurational theory by calibrating conditions as fuzzy sets and systematically examining how different combinations are sufficient or necessary for an outcome (Ragin, 2000; Ragin, 2008). Compared with regression-based models, fsQCA is particularly suited to the study of EGB, employees’ behaviors emerge from the interaction of institutional pressures, personal motivations, and capability states. Prior research has highlighted the role of institutional isomorphism in shaping behavior (DiMaggio and Powell, 1983), intrinsic and extrinsic motivations (Ryan and Deci, 2000), and individual efficacy beliefs (Bandura, 1997). Moreover, studies of ecological responsiveness demonstrate that green behaviors often result from the joint influence of external pressures, internal motivations, and organizational capabilities (Bansal and Roth, 2000). By capturing multiple and equally valid pathways, fsQCA allows researchers to account for both compliance-driven and voluntary forms of green behavior.

### 2.4 Hypotheses development

In summary, this study proposes the following hypotheses:

*H*1: Different combinations of institutional pressures (ECSR, GTL, GPM), capability states (GSE, JA), and motivational drivers (GV) are sufficient to generate task-oriented green behavior (TGB) as well as voluntary green behavior (VGB).

*H*2: The combinations of conditions that lead to the presence of TGB or VGB differ from those that lead to their absence.

*H*3: Employee green behavior (EGB) results from distinct Drive–State–Pressure configurations, with some configurations producing compliance-based TGB and others producing discretionary VGB.

**Figure 1:**
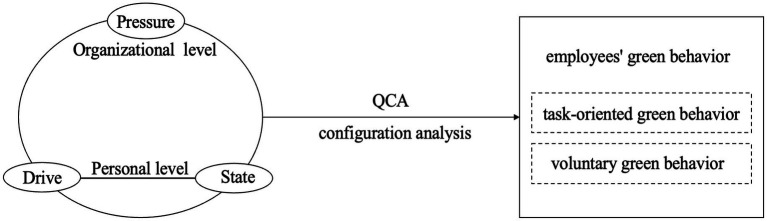
Conceptual model.

Taken together, these hypotheses reflect the configurational nature of employee green behaviors within the DSP framework. The overall conceptual model is presented in Figure 1.

## 3 Research method

### 3.1 Data collection and respondents

From June 13 to June 17, 2024, online questionnaires were distributed to hotel industry employees in China via the Credamo platform. The sample focused on large-chain hotels across 23 provincial-level regions, including Beijing, Shanghai, and Nanjing. Of the 398 questionnaires distributed, 356 valid responses were retained after excluding those that failed attention checks. The final sample consisted of 228 females (64.0%) and 128 males (36.0%), with 67.7% holding a bachelor’s degree. Respondents had an average age of 32.08 years (SD = 8.21) and an average organizational tenure of 5.34 years (SD = 4.07).

### 3.2 Instrument

Data collection was conducted using a structured questionnaire. Employee green behavior (EGB) was measured with Bissing-Olson et al. (2013) 6-item scale, which differentiates between task-oriented green behavior (TGB; e.g., “I complete assigned tasks using environmentally friendly methods”) and voluntary green behavior (VGB; e.g., “I proactively initiate workplace environmental initiatives”). Responses were recorded on a five-point Likert scale, demonstrating high reliability (*α* = 0.862 for TGB; α = 0.899 for VGB).

Green values were measured using eight personal norm items adapted from Steg et al. (2005), demonstrating excellent internal consistency (α = 0.916). Green self-efficacy was assessed with Chen et al.’s (2015) 6-item scale (α = 0.889). Job autonomy was measured using Spreitzer’s (1995) 3-item scale (α = 0.834). Green performance management (GPM) was evaluated through four objective-assessment items from Dumont et al. (2017), demonstrating strong reliability (α = 0.900). Environment-oriented CSR (E-CSR) was assessed using the first four items from Farooq et al.’s (2017) scale (α = 0.843). Green transformational leadership (GTL) was operationalized through the green intellectual stimulation and personalized care subscales from Robertson’s (2018) 12-item instrument (α = 0.896). All measurement items are presented in the Appendix A.

### 3.3 NCA

Scholarly discourse often conflates necessity and sufficiency, though they are distinct (Chung, 1969). Necessary causality means an outcome cannot occur without a condition (“no X, no Y”), while sufficient causality means a condition alone ensures the outcome (“if X, then Y”; Dul, 2016). This study employs Necessary Condition Analysis (NCA) and Qualitative Comparative Analysis (QCA) to disentangle these logics. NCA assesses whether the six DSP model factors—green values, green self-efficacy, job autonomy, green performance management, environment-oriented CSR, and green transformational leadership—are necessary for task-oriented (TGB) and voluntary green behavior (VGB), while QCA identifies sufficient causal configurations. The NCA was implemented in R (version 4.3.3) using the NCA package (version 4.0.1).

### 3.4 Bottom-up QCA

We employed fuzzy-set Qualitative Comparative Analysis (fsQCA) for two main reasons. First, fsQCA enables the exploration of equifinality, uncovering multiple and equally valid pathways that lead to employees’ green behaviors (Ragin, 2008; Fiss, 2011). Second, it accounts for causal asymmetry, recognizing that the conditions sufficient for the presence of task-oriented or voluntary green behaviors may not simply be the inverse of those leading to their absence (Ma et al., 2022). These features make fsQCA especially valuable for studying complex behavioral phenomena in organizational contexts.

However, adequacy analysis in QCA often encounters challenges regarding sufficiency, necessity, and completeness, as traditional top-down approaches face limitations such as rigid dichotomization and heavy reliance on counterfactuals. To overcome these issues, Ding (2023) proposed a bottom-up QCA method with four key innovations: (1) bypassing dichotomous truth table constraints, (2) minimizing counterfactual dependence, (3) scaling efficiently without inflating complexity, and (4) reducing omitted variable bias while ensuring validity. By generating parsimonious solutions through algorithmic iteration, this approach enhances rigor, replicability, and causal inference reliability in complex behavioral studies. In line with these methodological advancements, the configurational analyses in this study were conducted in R (version 4.3.3) using the QCA package (version 3.22), which enables robust and transparent implementation of fsQCA procedures.

### 3.5 Variable calibration

In fsQCA, the raw data need to be calibrated into fuzzy-set membership scores ranging from 0 to 1, which represent the degree to which a case belongs to a given condition. A score of 1 indicates full membership (the condition is at a high level), 0 indicates full non-membership (the condition is at a low level), and 0.5 represents the point of maximum ambiguity (the crossover point), where it is difficult to determine whether the condition is present or absent. Following prior methodological recommendations (Crilly et al., 2012), values that were exactly calibrated at 0.5 were adjusted slightly to 0.5001 to prevent these cases from being excluded during the truth table analysis.

We adopted an internal calibration strategy, meaning that the thresholds for high and low levels of each condition were defined relative to the distribution of the study sample rather than absolute external standards (Pappas et al., 2017). Specifically, for each condition we computed the sample mean and standard deviation, setting the mean as the crossover point (membership = 0.5). Full membership (membership = 1.0) was anchored at one standard deviation above the mean, while full non-membership (membership = 0.0) was anchored at one standard deviation below the mean. This parametric approach is widely used in organizational behavior research, where defining “high” or “low” levels in relative rather than absolute terms is more meaningful (Aiken et al., 1991). For example, an employee who scores 5 on a 7-point scale may appear high in absolute terms, but in comparison with peers in the same organization, this level could be relatively low. Thus, internal calibration captures the contextual nature of behavioral tendencies more appropriately.

The complete calibration anchor points for all antecedent conditions and outcome variables are presented in Table 1. For ease of interpretation, following prior studies (Xing et al., 2025; Ma et al., 2022), we refer to conditions or outcomes above the crossover point as “high” (presence of condition/outcome) and those below as “low” (absence of condition/outcome) throughout the results section.

**Table 1:** Calibration anchors for each fuzzy set.

Sets	Calibration anchors		
	Fully in	Crossover	Fully out
GV	4.91	4.34	3.78
GSE	4.82	4.21	3.59
JA	4.75	4.02	3.29
GPM	4.59	3.67	2.75
ECSR	4.76	4.04	3.33
GTL	4.73	4.00	3.27
TGB	4.92	4.28	3.65
VGB	4.89	4.20	3.52

GV, green values; GSE, green self-efficacy; JA, job autonomy; GPM, green performance management; ECSR, environment-oriented CSR; GTL, green transformational leadership; TGB, task-oriented green behavior; VGB, voluntary green behavior.

## 4 Analysis

### 4.1 Reliability and validity analysis

All constructs were measured using multi-item scales adapted from prior validated research (e.g., Bissing-Olson et al., 2013). Although no pilot test was conducted prior to the formal survey, the adoption of well-established scales helped ensure measurement reliability and validity. To further assess the psychometric properties of the instruments, internal consistency reliability was examined using Cronbach’s alpha coefficients in SPSS 24.0, with all constructs demonstrating excellent reliability (*α* > 0.80), exceeding the recommended threshold of 0.70 (Nunnally, 1978). In addition, confirmatory factor analysis (CFA) was conducted in AMOS 24.0 to evaluate construct validity. As shown in Table 2, the CFA results confirmed a good model fit (CFI = 0.95, TLI = 0.93, RMSEA = 0.06), with all factor loadings statistically significant (*p* < 0.001), thereby establishing measurement robustness. We further examined discriminant validity using the Fornell–Larcker criterion. As shown in Table 3, the square roots of AVEs (on the diagonal) are greater than the correlations between constructs (off-diagonal), indicating satisfactory discriminant validity.

**Table 2:** Overall model fitting results.

Items	Estimate	AVE	CR
GV1	0.743	0.5943	0.9206
GV2	0.708		
GV3	0.882		
GV4	0.685		
GV5	0.711		
GV6	0.908		
GV7	0.739		
GV8	0.76		
GSE1	0.745	0.5904	0.8953
GSE2	0.788		
GSE3	0.725		
GSE4	0.682		
GSE5	0.929		
GSE6	0.716		
JA1	0.806	0.6301	0.8359
JA2	0.736		
JA3	0.836		
GPM1	0.82	0.6937	0.9005
GPM2	0.833		
GPM3	0.855		
GPM4	0.823		
ECSR1	0.752	0.5755	0.8443
ECSR2	0.781		
ECSR3	0.753		
ECSR4	0.748		
GTL1	0.767	0.5937	0.8976
GTL2	0.778		
GTL3	0.789		
GTL4	0.745		
GTL5	0.753		
GTL6	0.79		
CMIN/DF	RMR	GFI	CFI
1.449	0.029	0.906	0.971

AVE, Average Variance Extraction; CR, Consistency Ratio; RMR, Root Mean Square Residual; GFI, Goodness of Fit Index; CFI, Comparative Fit Index.

**Table 3:** Correlation matrix and discriminant validity.

	ECSR	GPM	GSE	GV	GTL	JA
ECSR	0.7586					
GPM	0.248**	0.8329				
GSE	0.412**	0.266**	0.7684			
GV	0.434**	0.368**	0.595**	0.7709		
GTL	0.299**	0.252**	0.482**	0.565**	0.7705	
JA	0.267**	0.254**	0.438**	0.543**	0.368**	0.7938
AVE	0.5755	0.6937	0.5904	0.5943	0.5937	0.6301

* indicates <0.05, ** indicates <0.01. ECSR, environment-oriented CSR; GPM, green performance management; GSE, green self-efficacy; GV, green values; GTL, green transformational leadership; JA, job autonomy.

### 4.2 Necessary conditions analysis

Necessary Condition Analysis (NCA) identifies necessary conditions by assessing the effect size (d) and statistical significance of antecedent variables, with necessity levels further quantified through bottleneck analysis. As shown in Tables 4, 5, the NCA results for task-oriented green behavior (TGB) and voluntary green behavior (VGB) indicate that no individual condition meets the necessity threshold (d > 0.1, *p* < 0.05) as per Dul et al. (2020) criteria. The analysis utilized two estimation methods: ceiling regression (CR) for continuous variables and ceiling envelope (CE) for discrete variables. Given the absence of necessary conditions for both behavioral types, bottleneck level analysis was deemed unnecessary.

**Table 4:** Analysis on the necessity of NCA to individual conditions of employees’ task-oriented green behavior.

Condition^(1)^	Method	Accuracy	Effect size^(d)^	*p*-value^(2)^
GV	CR	100%	0.002	0.014
	CE	100%	0.004	0.014
GSE	CR	99.4%	0.008	0.000
	CE	100%	0.010	0.005
JA	CR	100%	0.000	1.000
	CE	100%	0.000	1.000
GPM	CR	100%	0.000	1.000
	CE	100%	0.000	1.000
ECSR	CR	100%	0.000	1.000
	CE	100%	0.000	1.000
GTL	CR	100%	0.000	1.000
	CE	100%	0.000	1.000

(1) Use the membership value of the calibrated fuzzy set; (2) permutation test is adopted, and the number of repeated sampling is 10,000. (d) Refers to the effect size in Necessary Condition Analysis (NCA).

**Table 5:** Analysis on the necessity of NCA to individual conditions of employees’ voluntary green behavior.

Condition^(1)^	Method	Accuracy	Effect size^(d)^	*p*-value^(2)^
GV	CR	100%	0.002	0.045
	CE	100%	0.003	0.045
GSE	CR	100%	0.000	1.000
	CE	100%	0.000	1.000
JA	CR	100%	0.000	1.000
	CE	100%	0.000	1.000
GPM	CR	100%	0.000	1.000
	CE	100%	0.000	1.000
ECSR	CR	100%	0.000	1.000
	CE	100%	0.000	1.000
GTL	CR	100%	0.000	1.000
	CE	100%	0.000	1.000

(1) Use the membership value of the calibrated fuzzy set; (2) permutation test is adopted, and the number of repeated sampling is 10,000. (d) Refers to the effect size in Necessary Condition Analysis (NCA).

### 4.3 Data analysis and results

The fsQCA results were reported using the configurational notation system, as established and shown in Tables 6, 7. In these tables, filled circles (●) indicate the presence of a condition, crossed circles (⊗) signify its absence (Fiss, 2011), and blank cells represent logical remainders (i.e., conditions irrelevant to the causal recipe). This symbolic framework facilitates a clear visualization of core and peripheral conditions across sufficient configurations while ensuring methodological consistency with leading QCA studies.

**Table 6:** Configurations for employees’ implementing a high level of task-oriented green behavior.

Belonging dimension	Antecedent conditions	S1	S2	S3	S4	S5	S6	S7	S8
Drive	GV	●		●			●		●
State	GSE					●	●	●	
	JA		●						●
Pressure	ECSR			●	●	●			
	GPM			●	●		●	●	●
	GTL	●	●		●	●		●	
	InclS	0.751	0.765	0.76	0.777	0.774	0.777	0.784	0.764
	PRI	0.61	0.638	0.61	0.638	0.606	0.617	0.633	0.618
	COVS	0.592	0.613	0.432	0.439	0.436	0.413	0.426	0.457
	InclS	0.688							
	PRI	0.558							
	COVS	0.829							

●, presence of the condition; ⊗, absence of the condition; blank space, condition is irrelevant in the configuration.

**Table 7:** Configurations for employees’ implementing a high level of voluntary green behavior.

Belonging dimension	Antecedent conditions	S1	S2	S3
Drive	GV		⊗	
State	GSE	●		⊗
	JA			●
Pressure	GPM	⊗	⊗	
	ECSR	●	●	
	GTL			⊗
	InclS	0.815	0.825	0.832
	PRI	0.615	0.608	0.605
	COVS	0.32	0.287	0.288
	InclS	0.767		
	PRI	0.565		
	COVS	0.476		

●, presence of the condition; ⊗, absence of the condition; blank space, condition is irrelevant in the configuration.

#### 4.3.1 High-level task-oriented green behavior-driven path

Table 6 identifies eight pathways to high-level task-oriented green behavior (TGB), grouped into two frameworks: the Pressure-Dominant Path and the Agency-Pressure Synergy Path. The latter includes three subtypes: Single Agency-Mimetic Pressure, Agency-Composite-Coercive Pressure Synergy, and Composite Pressure-Single Agency Adaptation.

Across these pathways, the core statistical indicators—Inclusion Score (InclS), Proportional Reduction in Inconsistency (PRI), and Coverage Score (COVS)—demonstrate strong model consistency and explanatory power. The InclS values range from 0.751 to 0.784, indicating that each pathway accounts for approximately 75.1 to 78.4% of cases achieving high-level TGB, signifying robust explanatory strength. The PRI values range from 0.606 to 0.638, confirming that the identified configurations consistently contribute to TGB, although some behavioral variation arises due to contextual factors. The COVS values, ranging from 0.413 to 0.613, suggest moderate to high coverage, indicating that while each pathway plays a crucial role in explaining green behaviors, additional mechanisms may also contribute to observed TGB outcomes.

Category 1: Pressure–Dominant Path

The Pressure-Dominant Path (S1) demonstrates that a three-dimensional institutional matrix—coercive (GPM), normative (ECSR), and mimetic (GTL) pressures—can substitute for individual agency to drive high-level task-oriented green behaviors (TGB). Coercive pressure (GPM) enforces compliance through reward–punishment mechanisms, embedding green behaviors into formal controls (Juma and Moronge, 2015). Normative pressure (ECSR) reshapes cognitive schemas, internalizing green practices as professional ethics (Glavas and Kelley, 2014). Mimetic pressure (GTL) provides behavioral scripts, guiding employees with low green self-efficacy through role modeling (DiMaggio and Powell, 1983). Together, these pressures create a nested institutional system where TGB becomes an identity-driven practice rather than an imposed obligation.

This mechanism underscores how organizational pressure systems facilitate task-oriented green behaviors through an “institutional substitution effect,” transcending the motivation–ability paradigm. Effective management requires a multidimensional pressure synergy: integrating green performance indicators into appraisals for coercive isomorphism, leveraging CSR narratives for normative internalization, and using leadership exemplars to reinforce mimetic influence. Aligning these mechanisms embeds sustainability into employee behavior, fostering a deep-rooted green culture beyond mere compliance.

Category 2: Agency–Pressure Synergy Path
Single Agency–Mimetic Pressure–Driven Path

In S2a, employees’ intrinsic green values, combined with mimetic pressure from green transformational leadership, drive high-level task-oriented green behaviors. In S2b, even when the driving force comes from job autonomy rather than intrinsic values, mimetic pressure still facilitates the achievement of high-level task-oriented green behaviors. Both paths illustrate a compensatory coupling mechanism in which mimetic pressure complements distinct agency elements. In S2a, deep-seated green values motivate employees to align with organizational environmental goals through the self-consistency mechanism. At the same time, green transformational leadership provides proximal behavioral cues through role modeling and symbolic interaction (Farrukh et al., 2022), forming a dual-channel activation system of “value-driven behavior modeling.” This synergy reinforces self-concept and enables green behaviors to transcend inherent ability limitations.

In S2b, job autonomy provides employees with the decision-making space needed for behavior regulation (Wan et al., 2024), while green transformational leadership offers a replicable practice template (Weiss, 1977). This synergy simplifies the execution path by translating abstract environmental norms into concrete guidelines, thereby compensating for limited self-efficacy and facilitating efficient task-oriented green behaviors. The compensatory function of mimetic pressure in both paths illustrates the equifinality principle (Gresov and Drazin, 1997), demonstrating that different agency–pressure configurations—GV combined with GTL in S2a and JA combined with GTL in S2b—can lead to equivalent task-oriented green behavior outcomes. This observation aligns with institutional theory, which suggests that mimetic processes reduce uncertainty by providing socially sanctioned “recipes” for action.

Agency–Composite–Coercive Pressure Synergy Path

In the Agency–Composite–Coercive Pressure Synergy Paths (S3a–S3b), a fundamental shift in institutional logic is evident. Unlike mimetic pressure, coercive pressure, represented by green performance management (GPM), lacks an intrinsic meaning-making function. As a result, it requires the complementarity of agency elements—such as the combination of GV and GSE (S3a) or GV and JA (S3b)—to counterbalance its psychological costs. For instance, in S3a, the interplay between GV, GSE, and GPM enables a more constructive response to coercive pressure. When employees possess strong GSE, they are less likely to experience negative emotional reactions to GPM. Instead, they perceive coercive environmental mandates as opportunities to enhance self-management and actively engage in decision-making, rather than merely as external constraints (Deci and Ryan, 2012). This triadic synergy underscores that coercive pressure enhances task-oriented green behaviors (TGB) only when it is reinforced by agency factors that provide both intrinsic motivation and the operational competence needed to navigate organizational demands.

The critical factor in this dynamic is the complementary interplay between coercive pressure and agency elements. A single agency factor alone is insufficient to counterbalance the psychological costs associated with coercive pressure. Instead, an effective response requires a dual configuration—combining motivational drivers (green values) with state-related elements (such as green self-efficacy and job autonomy). In this mechanism, green values mitigate the external attribution tendencies triggered by coercive demands, fostering a sense of personal commitment rather than external obligation. Simultaneously, state-related elements provide the necessary psychological capital for execution, ensuring that institutional pressure is effectively translated into concrete behavioral outcomes. Compared with the “single-agency-mimetic-driven” path—where mimetic pressure alone activates the behavior chain—coercive pressure, due to its inherent lack of meaning-making capacity, necessitates a complementary agency combination to achieve both value internalization and path simplification.

Composite Pressure–Single Agency Adaptation Path

This situation encompasses three paths—S4a, S4b, and S4c—that facilitate high-level task-oriented green behaviors. In S4a, employees’ green values, combined with normative pressure from environment-oriented CSR and coercive pressure from green performance management, effectively drive TGB. In this process, CSR reshapes employees’ cognitive frameworks to institutionalize green values (Glavas and Kelley, 2014), while performance management establishes a behavioral baseline (Juma and Moronge, 2015). Together, they create a dual assurance mechanism that converts composite institutional pressure into a structured value-realization channel, reducing cognitive dissonance and reinforcing both value identification and institutional compliance.

In S4b, a single ability factor—green self-efficacy—works in tandem with normative pressure from CSR and mimetic pressure from green transformational leadership. In this scenario, CSR establishes a cognitive benchmark, while leadership demonstrates the practical feasibility of green practices (Glavas and Kelley, 2014), creating a reinforcing effect that continuously strengthens self-efficacy. This process enables a smooth transition from ability validation to the internalization of organizational norms, thereby fostering TGB.

In S4c, even when green self-efficacy is the sole agency element, its combination with coercive and mimetic pressures ultimately leads to high-level TGB. Specifically, green performance management enforces result control, reinforcing the necessity of the behavior (Juma and Moronge, 2015), while green transformational leadership simplifies execution through process demonstrations. Under these conditions, green self-efficacy serves as a buffering mechanism, allowing employees to reinterpret coercive institutional demands as opportunities to showcase and further develop their capabilities, ultimately driving behavioral transformation.

In both Pressure-Dominant and Agency-Pressure Synergy models, institutional pressures compensate for the limitations of individual agency. Normative pressure legitimizes values, coercive pressure sets behavioral baselines, and mimetic pressure ensures feasibility. This supports DiMaggio and Powell (1983) isomorphism framework, demonstrating how structured pressures enhance agency efficiency. While mimetic pressure can activate green behavior with a single agency factor, coercive pressure requires complementary agency elements for value internalization and execution. Ultimately, a multidimensional pressure synergy network offers a more stable and effective approach to fostering sustainable workplace behaviors.

#### 4.3.2 High-level voluntary green behavior-driven path

This study identifies three distinct pathway combinations leading to high-level voluntary green behaviors (VGB) among hotel employees: the Efficacy-Norm Compensation Path (S1), the Singular Pressure Breakthrough Path (S2), and the Autonomy-Institution Synergy Path (S3). These pathways illustrate how organizational pressures and individual agency elements interact to foster voluntary green engagement in the absence of explicit mandates. Across these three paths, the InclS values range from 0.815 to 0.832, indicating that each pathway accounts for 81.5 to 83.2% of cases exhibiting high-level VGB, underscoring the strong explanatory power of the model. The PRI values, ranging from 0.605 to 0.615, suggest that while these pathways reliably predict voluntary green behavior, slight variations in behavioral expression may arise due to contextual factors. The COVS values, which range from 0.287 to 0.32, indicate that these configurations explain a moderate proportion of observed voluntary green behaviors. This suggests that while these pathways play a significant role in shaping VGB, additional influencing factors may also be at play.

In Path 1, green self-efficacy enhances internal motivation (Tabernero and Hernández, 2011), while environment-oriented CSR (ECSR) shapes organizational identity (Glavas and Kelley, 2014), creating a dual-motivation mechanism. Self-efficacy strengthens agency, and ECSR provides meaning. Notably, the absence of coercive pressure enhances self-determination, allowing normative pressure to shift from constraint to value resonance. Green self-efficacy plays a crucial role in this internalization process by reducing uncertainty and reinforcing commitment to sustainable actions (Saleem et al., 2024).

In Path 2, environment-oriented CSR reshapes employees’ social identity cognition through institutional narratives (Glavas and Kelley, 2014), creating an “organizational identity transfer” effect. In this process, corporate environmental responsibility is reframed as an extension of employees’ professional roles, fostering deeper behavioral commitment (Robertson and Barling, 2013). The absence of coercive pressure plays a key regulatory role by preventing cognitive overload that could arise from conflicting institutional and value-based mandates. Without coercive enforcement, employees experience low-resistance internalization, where CSR-driven descriptive norms (observing others’ behaviors) and injunctive norms (perceived expectations) jointly shape green behavioral scripts. This mechanism aligns with the dual-process model of social information processing (Chaiken, 1980), in which organizational CSR simultaneously conveys both what is done and what should be done.

In Path 3, job autonomy activates employees’ innovative agency by providing opportunities for job crafting. This compensatory mechanism allows employees to explore alternative green practices, even in the absence of explicit self-efficacy cues. The lack of mimetic pressure serves as a behavioral catalyst, encouraging employees to deviate from conventional templates and reinterpret institutional expectations through personalized strategies. In other words, without traditional reinforcement elements—such as leadership modeling or efficacy-based motivation—the cognitive flexibility enabled by job autonomy allows employees to reconstruct environmental problem-solving approaches (Wan et al., 2024). This transformation reframes institutional constraints as opportunities for self-challenge. These findings emphasize the strategic role of selective institutional pressures and agency-driven mechanisms in shaping voluntary green behaviors, highlighting that no single pathway dominates. Instead, a dynamic interplay of factors enables employees to engage in sustainable practices beyond formal mandates.

## 5 Conclusion, implication and limitation

### 5.1 Conclusion and discussion

Drawing on the DSP framework and configurational approach, this study investigated how institutional pressures (ECSR, GTL, GPM), capability states (GSE, JA), and motivational drivers (GV) combine to shape task-oriented and voluntary green behaviors. Consistent with our hypotheses, the results confirm that employee green behaviors emerge not from isolated predictors but from distinct configurations of antecedent conditions, thereby demonstrating the principles of equifinality and causal asymmetry.

Our findings align with prior research in showing that institutional pressures are critical in shaping compliance-oriented behaviors, while personal values and efficacy underpin discretionary engagement (Wang et al., 2018). At the same time, the results deviate from traditional perspectives that portray external pressures solely as restrictive. We find that under certain configurations, the selective absence of pressure can encourage agency-driven innovation, offering a more nuanced understanding of how organizations can foster voluntary green behaviors (Zafar et al., 2022).

By mapping multiple viable pathways to both TGB and VGB, this study extends configurational theorizing in organizational behavior. It demonstrates that sustainability engagement cannot be reduced to single “critical factors” but instead arises from diverse pressure–state–drive combinations. This insight contributes theoretically by bridging institutional and motivational perspectives, and practically by suggesting that managers should design adaptive systems: structured pressure regimes to secure task compliance and enabling environments that cultivate autonomy and intrinsic motivation for voluntary engagement.

In conclusion, this study highlights that organizational green behavior is not governed by one dominant mechanism but emerges through multiple, equally effective pathways. Recognizing this diversity helps move beyond linear models toward a more realistic understanding of how institutions and individuals interact to advance sustainability.

### 5.2 Theoretical implications

This study advances environmental behavior research through three key theoretical contributions, each critically engaging with existing literature while offering novel insights into the interplay between organizational systems and individual agency. First, this research bridges the macro–micro divide in institutional theory by clarifying how organizational pressures shape individual green behaviors. While institutional theory emphasizes coercive, normative, and mimetic pressures as drivers of isomorphism (Jaja et al., 2019), it rarely explores their employee-level impact. The DSP model fills this gap by mapping pressures to behavioral pathways: coercive mechanisms like green performance management (GPM) standardize task-oriented green behaviors (TGB), while normative pressures, such as environment-oriented CSR (ECSR), foster voluntary green behaviors (VGB) by embedding sustainability into corporate identity. Mimetic influences, exemplified by green transformational leadership (GTL), legitimize green experimentation through role modeling. Crucially, the study highlights negotiated institutionalization, where employees reinterpret organizational pressures through intrinsic green values (GV) and self-efficacy (GSE). TGB aligns with structured mandates, reinforcing compliance through coercive and normative pressures, whereas VGB emerges from agentic negotiation, as employees internalize normative CSR cues or mimetic leadership influences to integrate sustainability into their role identity (Steg and Vlek, 2009). This refines institutional theory by demonstrating that organizational pressures are not static constraints but dynamically shaped through employee agency.

Second, this study addresses methodological limitations in green behavior research by demonstrating the value of configurational thinking. Traditional symmetric methods, such as regression analysis, assume linear, additive relationships between variables, which can obscure the complexity of behavioral causation (Breiman, 2001). In contrast, our fuzzy-set Qualitative Comparative Analysis (fsQCA) reveals equifinality and substitution effects that challenge conventional wisdom. For instance, the identification of eight distinct pathways to high task-oriented green behavior (TGB) dismantles the myth of a universal “best practice” showing that compliance can emerge from moral alignment through green values and transformational leadership, institutional control through green performance management and environmental CSR, or capability scaffolding through green self-efficacy and performance management. Equally significant is the finding that cultural pressures, such as environmental CSR, can substitute for intrinsic green values in driving voluntary green behaviors (VGB). In contrast, task-oriented green behaviors (TGB) require a structured pressure-agency alignment, where leadership modeling plays a critical role in amplifying the effects of green values. When transformational leadership is present, green values function as a reinforcing rather than an indispensable factor, aligning with Bissing-Olson et al.’s (2013) argument that values alone are insufficient but gain influence through social validation. This explains why certain TGB pathways rely on leadership to enhance compliance-driven engagement, ensuring that green practices are embedded within employees’ professional norms. These insights validate Fiss (2011) advocacy for set-theoretic methods in behavioral research, shifting the paradigm from linear causality to context-dependent behavioral configurations.

Third, this research clarifies paradoxes in environmental psychology by distinguishing task-oriented and voluntary green behaviors. Prior studies often conflate these, leading to contradictory findings (Wiernik et al., 2016). For instance, green performance management reinforces task-oriented green behaviors (TGB) through clear expectations and accountability (Ruepert et al., 2016) but may hinder voluntary green behaviors (VGB) by dampening intrinsic motivation (Davis et al., 2020). This reflects the compliance-innovation paradox, where institutional pressures ensure consistency in structured tasks but constrain discretionary initiatives requiring autonomy. In contrast, VGB relies on facilitative mechanisms, with normative pressures like environmental CSR acting as cultural anchors rather than rigid mandates (Matten and Moon, 2008). This fosters personal agency through green values, self-efficacy, and job autonomy, aligning with self-determination theory (Deci and Ryan, 2012): coercive pressures enhance compliance but undermine autonomy essential for innovation-driven sustainability. Similarly, the values-action gap—where green values alone fail to predict environmental behaviors (Steg and Vlek, 2009)—is reframed as a configurational mismatch. Green values require activation through leadership modeling or cultural reinforcement via CSR to translate pro-environmental attitudes into action.

This behavioral typology enhances theoretical precision by outlining the specific pathways through which green behaviors emerge. It also offers practical clarity, explaining why standardized sustainability initiatives often fail to accommodate the diverse motivational structures that drive different types of green behaviors.

### 5.3 Managerial implications

For organizations navigating sustainability transitions, our findings advocate a tripartite strategic realignment that integrates behavioral science principles with HR architecture redesign. First and foremost, organizations must tailor interventions to the distinct motivations behind task-oriented (TGB) and voluntary green behaviors (VGB). For TGB, a pressure-capacity synergy should integrate green performance management (GPM) with quantifiable targets and green transformational leadership (GTL) training to reinforce compliance. In contrast, VGB requires autonomy-driven motivation. Environment-oriented CSR should go beyond compliance, embedding sustainability into daily experiences (Adu-Gyamfi et al., 2021)—e.g., linking hotel waste reduction to local ecosystem restoration. Participatory initiatives like gamified eco-challenges, employee-led green innovation labs, and “green experimentation zones” can further enhance intrinsic motivation and job autonomy, fostering engagement and creativity.

Secondly, leadership development programs must align with organizational work design (Hanson, 2013) by tailoring green influence strategies to different workplace contexts. In high-autonomy departments, where employees have greater behavioral discretion, managers should act as cultural facilitators (Teräväinen and Junnonen, 2019), fostering a green innovation culture through shared vision-building and participatory decision-making. In contrast, in standardized operational units with rigid procedural constraints, managers should function as process optimizers, embedding sustainability seamlessly into workflow efficiency through structured task alignment and behavioral nudges.

Finally, a systemic HR architecture must institutionalize a dual-reinforcement approach, ensuring that institutional pressures—coercive, normative, and mimetic—are operationalized through structured HR mechanisms. First, competency models should prioritize green self-efficacy (GSE) development through VR-enabled sustainability simulations, transforming abstract environmental principles into hands-on operational skills. Second, organizations must integrate green behaviors into formal appraisal systems (coercive pressure; Saeed et al., 2019) while fostering peer-driven sustainability incentives (mimetic pressure), such as interdepartmental eco-challenges and public recognition programs. This structured yet flexible approach ensures that green behaviors are not merely encouraged but become deeply ingrained in employees’ professional identity.

### 5.4 Limitations and future research directions

While this study provides an in-depth understanding of employee green behavior, it has certain limitations. First, the study is based on a cross-sectional dataset of hotel employees in mainland China. This specific cultural and industry context may shape how employees respond to institutional pressures. For example, employees in China may react differently to coercive pressures compared to those in Western contexts. Thus, the generalizability of our findings is limited. Future studies could conduct cross-cultural and cross-industry comparisons to validate and extend our conclusions.

Second, although the Drive-State-Pressure (DSP) model effectively captures the interaction between intra-organizational institutional and individual factors, it does not directly incorporate external pressures such as customer environmental expectations and government regulations. Future research should integrate these external influences to provide a more holistic understanding of the drivers of green behavior.

Third, the study employs fuzzy-set Qualitative Comparative Analysis (fsQCA), which identifies multiple behavioral pathways but is sensitive to threshold calibration and sample composition. To mitigate these issues, future research could combine fsQCA with longitudinal case studies, enabling the dynamic evolution of green behaviors to be more fully captured.

Fourth, organizational and individual contextual factors—such as company-level green norms and employees’ career stages—were not fully addressed. For instance, early-career employees may be more influenced by mimetic pressures, whereas senior employees may rely more on intrinsic motivation and job autonomy. Exploring these dynamics could further enrich our understanding of green behavior across diverse contexts.

Fifth, the imbalance between female and male participants in the sample may affect the extent to which the results can be generalized to a broader workforce. Future studies should aim to include a more balanced gender distribution to validate and extend the findings.

## Data Availability

The raw data supporting the conclusions of this article will be made available by the authors, without undue reservation.

## Appendix A: Measurement items

**Table:**

Construct	Items (5-point Likert scale: 1 = strongly disagree, 5 = strongly agree)	Source
Green Values (GV)	I feel personally obligated to save as much energy as possible. I believe that, regardless of what others do, I have a moral obligation to save energy. I feel guilty when I waste energy. I feel a moral obligation to use environmentally friendly energy instead of conventional electricity. I should do everything I can to reduce my energy use. If I need to buy a new washing machine, I feel I should choose an energy-saving one. I believe I have a duty to protect the environment and nature in my daily behavior. If I save energy, I will become a better person.	Steg et al. (2005)
Green Self-Efficacy (GSE)	I can successfully practice environmental protection concepts. I can achieve most of my environmental goals. I can effectively handle environmental tasks. I can effectively carry out environmental responsibilities. I can solve environmental problems. I can find creative ways to solve environmental problems.	Chen et al. (2015)
Job Autonomy (JA)	I have a great deal of autonomy in deciding how I do my work. I can decide on my own how to go about doing my work. I have considerable opportunity for independence and freedom in my work.	Spreitzer (1995)
Green Performance Management (GPM)	The company sets environmental goals for employees. The company takes employees’ workplace green behavior into account in performance evaluations. The company links employees’ workplace green behavior to rewards and compensation. The company considers employees’ workplace green behavior in promotion decisions.	Dumont et al. (2017)
Environment-oriented CSR (ECSR)	The company actively engages in activities to protect and improve the natural environment. The company invests to ensure that future generations can enjoy a better living environment. The company takes measures to reduce the negative impact of hotel operations on the natural environment. The company is committed to achieving sustainable development that is closely related to the well-being of future generations.	Farooq et al. (2017)
Green Transformational Leadership (GTL)	In my department, my immediate supervisor encourages me to think about environmental issues in different ways. In my department, my immediate supervisor is willing to accept my ideas for improving the company’s environmental performance. In my department, my immediate supervisor urges me to think creatively about how to improve our company’s environmental performance. In my department, my immediate supervisor recognizes my ability to enhance the company’s environmental performance. In my department, my immediate supervisor notices my personal contributions to the company’s environmental performance. In my department, my immediate supervisor is willing to spend time developing my environmental skills to promote our company’s environmental performance.	Robertson (2018)
Task-Oriented Green Behavior (TGB)	Today, I adequately completed assigned duties in environmentally-friendly ways. Today, I fulfilled responsibilities specified in my job description in environmentally-friendly ways. Today, I performed tasks that are expected of me in environmentally-friendly ways.	Bissing-Olson et al.’s (2013)
Voluntary Green Behavior (VGB)	Today, I took a chance to get actively involved in environmental protection at work. Today, I took initiative to act in environmentally-friendly ways at work. Today, I did more for the environment at work than I was expected to.	
